# Draft Genome Sequence of Mycoplasma wenyonii, a Second Hemotropic Mycoplasma Species Identified in Mexican Bovine Cattle

**DOI:** 10.1128/MRA.00875-18

**Published:** 2018-09-06

**Authors:** Rosa Estela Quiroz-Castañeda, Fernando Martínez-Ocampo, Edgar Dantán-González

**Affiliations:** a Unidad de Anaplasmosis, Centro Nacional de Investigación Disciplinaria en Parasitología Veterinaria, INIFAP, Jiutepec, Morelos, Mexico; b Laboratorio de Estudios Ecogenómicos, Centro de Investigación en Biotecnología, Universidad Autónoma del Estado de Morelos, Cuernavaca, Morelos, Mexico; University of California, Riverside

## Abstract

The hemotropic mycoplasma (hemoplasma) Mycoplasma wenyonii is an animal pathogen that affects bovine cattle health. Here, we present the draft genome sequence of the hemoplasma M. wenyonii strain INIFAP02 found in cattle from Mexico.

## ANNOUNCEMENT

Hemotropic mycoplasmas (hemoplasmas) are epierythrocytic bacteria that have been reported in cattle from several countries and may act synergistically during a coinfection (1–5). Animals infected with hemoplasmas show clinical signs that include severe anemia, ill thrift, fever, lymphadenopathy, depression, decreased milk production, infertility, and reproductive inefficiency (3, 6, 7). To date, “Candidatus Mycoplasma haemobos” and Mycoplasma wenyonii are the only reported hemoplasmas that infect cattle. Previously, we reported the draft genome sequence of “Ca. Mycoplasma haemobos” strain INIFAP01, the first genome sequence of this hemoplasma (8). Here, we report the draft genome sequence of M. wenyonii strain INIFAP02, an uncultivated hemoplasma identified in the blood of sick cattle from Chihuahua, Mexico.

Genomic DNA was obtained from blood samples of sick cattle using the UltraClean DNA BloodSpin kit (Mo Bio Laboratories) following the manufacturer’s instructions, and 2 µg of the genomic DNA was sequenced using the Illumina MiSeq system (2 × 300-bp paired-end reads). We obtained a total data set of 605,370 paired-end reads, of which 13,678 paired-end reads belonged to the genome of M. wenyonii strain INIFAP02. The remaining paired-end reads belonged to the genomes of bovine and intraerythrocytic microorganisms (data not shown). Illumina adapter sequences were removed using the ILLUMINACLIP trimming step of the Trimmomatic version 0.36 program (9). Poor-quality bases from the Illumina paired-end reads were removed with the DynamicTrim algorithm in the SolexaQA++ software package (10) using a Phred quality threshold (Q) of 13. The paired-end reads were *de novo* assembled using the SPAdes version 3.11.1 program (11) with the following options: (i) only runs assembly module (–only-assembler), (ii) reduce the number of mismatches (–careful), and (iii) *k*-mer lengths between 21 to 127. Contigs not belonging to the genus Mycoplasma were eliminated using the nucleotide collection (NR/NT) database of the Nucleotide BLAST (BLASTN) server (https://blast.ncbi.nlm.nih.gov/Blast.cgi) (12). The draft genome sequence was processed using the QUAST version 4.6.2 program (13). The draft genome of M. wenyonii strain INIFAP02 has 37 contigs with a total length of 596,665 bp, an *N*_50_ contig length of 35,044 bp, a G+C content of 33.43%, and a coverage of ∼7×.

We obtained the 16S rRNA gene sequence of M. wenyonii strain INIFAP02 using the RNAmmer version 1.2 server (http://www.cbs.dtu.dk/services/RNAmmer) (14). The 16S rRNA gene has a length of 1,480 bp and an alignment coverage between 99 and 100%, as well as 98, 96/95, and 82% identities with strains M. wenyonii Massachusetts, M. ovis Michigan (two sequences), and “Ca. Mycoplasma haemobos” INIFAP01, respectively (GenBank accession numbers CP003703, CP006935, and LWUJ01000012, respectively). These data confirm that strain INIFAP02 is closely related to the M. wenyonii species. The M. wenyonii strain INIFAP02 genome has 52% alignment coverage, 79% identity, and 53,563 fewer base pairs than the M. wenyonii Massachusetts genome (total lengths of 596,665 and 650,228 bp for strains INIFAP02 and Massachusetts, respectively) (15).

The draft genome sequence of M. wenyonii strain INIFAP02 was annotated automatically with the Rapid Annotations using Subsystems Technology (RAST) version 2.0 server (http://rast.nmpdr.org) (16), which identified 712 genes and 678 coding sequences.

### Data availability.

The whole-genome shotgun project of M. wenyonii strain INIFAP02 has been deposited in GenBank under the accession number QKVO00000000. The version described in this paper is the first version, QKVO01000000.
